# MicroRNAs regulate gene plasticity during cold shock in zebrafish larvae

**DOI:** 10.1186/s12864-016-3239-4

**Published:** 2016-11-15

**Authors:** I-Chen Hung, Yu-Chuan Hsiao, H. Sunny Sun, Tsung-Ming Chen, Shyh-Jye Lee

**Affiliations:** 1 Department of Life Science, National Taiwan University, Taipei, 10617 Taiwan; 2 Institute of Molecular Medicine, College of Medicine, National Cheng Kung University, Tainan, 70456 Taiwan; 3 Institute of Basic Medical Sciences, College of Medicine, National Cheng Kung University, Tainan, 70456 Taiwan; 4 Research Center for Developmental Biology and Regenerative Medicine, National Taiwan University, Taipei, 10617 Taiwan; 5 Research Center for Developmental Biology and Regenerative Medicine, National Taiwan University, Taipei, 10617 Taiwan; 6 Center for Biotechnology, National Taiwan University, Taipei, 10617 Taiwan; 7 Center for Systems Biology, Taipei, 10617 Taiwan

**Keywords:** Deep sequencing, miRNA, Clock genes, Zebrafish, Embryonic development, Cold stress

## Abstract

**Background:**

MicroRNAs (miRNAs) are critical regulators responding to acute environmental stresses in both plants and animals. By modulating gene expression, miRNAs either restore or reconstitute a new expression program to enhance cell tolerance to stresses. Cold shock is one of the stresses that can induce acute physiological responses and transcriptional changes in aquatic creatures. Previous genomic studies have revealed many cold-affected genes in fish larvae and adults, however, the role of miRNAs in acute cold response is still ambiguous. To elucidate the regulatory roles of miRNAs in the cold-inducible responses, we performed small RNA-seq and RNA-seq analyses and found potential cold regulatory miRNAs and genes. We further investigated their interactions and involvements in cold tolerance.

**Results:**

Small RNA-seq and RNA-seq identified 29 up-/26 down-regulated miRNAs and 908 up-/468 down-regulated genes, respectively, in responding to cold shock for 4 h at 18 °C. miRNA and transcriptomic analyses showed these miRNAs and mRNAs are involved in similar biological processes and pathways. Gene ontology enrichment analyses revealed the cold-induced genes were enriched in pathways, including melanogenesis, GnRH pathway, circadian rhythm, etc. We were particularly interested in the changes in circadian clock genes that affect daily metabolism. The enrichment of circadian clock genes was also observed in previous fish cold acclimation studies, but have not been characterized. To characterize the functional roles of circadian clock genes in cold tolerance, we individually overexpressed selected clock genes in zebrafish larvae and found one of the core clock genes *per2* resulted in better recovery from cold shock. In addition, we validated the interaction of *per2* with its associate miRNA, *dre-mir-29b*, which is also cold-inducible. It suggests the transcription of *per2* can be modulated by miRNA upon cold shock.

**Conclusions:**

Collectively, our observations suggest that miRNAs are fine turners for regulating genomic plasticity against cold shock. We further showed that the fine tuning of core clock gene *per2* via its associated miRNA, *dre-mir-29b*, can enhance the cold tolerance of zebrafish larvae.

**Electronic supplementary material:**

The online version of this article (doi:10.1186/s12864-016-3239-4) contains supplementary material, which is available to authorized users.

## Background

The survival of aquatic fish is constantly challenged by the changes in environmental factors, such as salinity, oxygen concentration, pH, osmotic pressure and temperature. The change in water temperature has a dramatic effect on physiology and behaviors of aquatic animals that it has been shown to be an “abiotic master factor” for fish [1, 2]. Cold shock is characterized as an acute reduction in ambient temperature that may cause rapid falling in body temperature and a cascade of physiological and behavioral responses [2]. The cold shock stress response in fish can be broadly grouped into three categories; primary, secondary, and tertiary responses [2–4]. The primary responses of cold shock rapidly change the neuroendocrine pathways, such as catecholamines and corticosteroids [5–7]. It is initiated by the central nervous system sensing low temperatures that stimulates the chromaffin cells to release catecholamines. The hypothalamus-pituitary-interrenal axis is then activated to secret corticotrophin-releasing hormone to stimulate adrenocorticotrophic hormone and subsequent release of cortisol [4, 8, 9]. Secondary response can be elicited by the primary response. The stress hormones and cortisol interact to induce glucose production in fish via glycogenolysis and glucogenesis [9, 10] that results in changes of protein expression, molecular responses, osmoregulatory, and immune systems [2, 11]. The tertiary responses are the global effects of cold shock to impair development rate, respiratory function, swimming behavior, and mortality [2].

Expressions of numerous genes are regulated by stresses in fish. Transcriptome analysis has been used in the discovery of many stress-inducible genes in cold - acclimated [12–14] or cold-shocked fishes [15, 16]. Those genes belong to a variety of biological processes, including chaperones, transcription factors, signal transduction pathways, metabolism, responses to stress or ion transporters like *kir2.2* channel in rainbow trout [17], *heat shock protein 70* in silver sea bream [18] and *glycerol*-*3*-*phosphate dehydrogenase* in rainbow smelt [19]. However, studies on non-model fishes have been difficult due to the lack of whole genome sequences and inability for genome manipulation. Zebrafish is a favorable model fish due to the availability of whole gene sequences and accessibility of genome manipulation. They can also be used for genomic research using high-throughput RNA-seq and small RNA-seq techniques. Moreover, zebrafish is a tropical animal acclimated to extreme temperature changes ranging from 6.2 to 39.2 °C in certain seasons [20]. This makes it an ideal model to investigate the regulatory mechanisms of temperature-dependent responses.

MicroRNAs (miRNAs) are short non-coding RNAs of around 22 nucleotides, which can modulate the stability and silence mRNAs [21]. A miRNA typically targets multiple mRNAs [22], which makes it a potential fine-tuner for different situations. Studies indicate that miRNAs play a key role in gene regulation in stress responses like oxidative reaction [23], temperature [24] or DNA damage stresses [25] in different organisms [26, 27]. However, relatively less is known about the functions of miRNA in aquatic species. *miR-8* and *miR-429* have been reported to be involved in the osmotic stress response in zebrafish [28, 29]. In contrast, high-throughput miRNAtomes analysis revealed no significant impact by miRNA-mediated gene regulation in the cold - acclimated adult zebrafish brains [30]. This result is puzzling, but we reasoned that the cold-induced responses might be different in organisms or tissues used. For example, fish larvae are more voluntary to environmental stresses like acute cold shock, but their responses to the changes in miRNA and transcriptomic profiles during acute cold shock remains unexplored.

Yang et al. claimed that miRNAs may have a minor role in regulating transcriptome plasticity in cold acclimated zebrafish brains based on the poor correlations between cold-induced transcriptomic and miRNAtome profiles [30]. This is in clear contrast to our previous understanding observed in other organisms [31, 32]. The discrepancy may be due to differences in organisms, tissue analyzed and experimental conditions. To further clarify this issue, we aimed to investigate the correlation between miRNAtome and transcriptome under cold shock in zebrafish larvae and to resolve possible cold-dependent regulatory mechanisms of selected miRNAs and their target genes. We discovered that similar biological processes and pathways were affected by cold in both miRNAtome and transcriptome analyses. In particular, we found notable correlations between circadian rhythm-related genes (known as circadian clock genes) [33] and their associate miRNAs. Core clock gene *per2* plays a major role in regulating circadian rhythm and daily metabolism [34]; we found that *dre-mir-29b* (*dre* denotes *Danio rerio*) can target *per2* in zebrafish larvae to possibly regulate cold shock response via modulating the expression of *per2*. More importantly, we demonstrated that the overexpression of *per2* in zebrafish larvae can increase their cold tolerance. These results indicate a pivotal role of miRNA in regulating gene plasticity to counter cold stress.

## Methods

### Fish maintenance

Zebrafish, *Danio rerio*, wild-type AB strain, were maintained under a 14 h light/10 h dark cycle at 28.5 °C. Embryos were collected by natural spawning and incubated in 0.3× Danieau’s buffer (1× Danieau’s buffer: 58 mM NaCl, 0.7 mM KCl, 0.4 mM MgSO_4_, 0.6 mM Ca(NO_3_)_2_, and 5.0 mM HEPES in double - distilled water, pH adjusted at 7.6) until examination or fixation.

### Cold shock and embryo collection

For cold shock experiment, 50 larvae were cultured at 28.5 °C until 96 h post fertilization (hpf), transferred into a 250 ml beaker containing 100 ml pre-chilled 0.3× Danieau’s buffer, and immediately incubated at designated temperatures until examination. The 96-hpf embryos before cold shock were collected as the control group. Embryos incubated at 28.5 °C or 18 °C for 4 more hours were collected as the normal or cold shock group, respectively. Two different batches of samples were collected for further next generation sequencing (NGS) analysis.

### miRNA library construction and sequencing

RNA samples were collected from the control, normal and cold shock groups for cDNA library construction. RNAs of desired time points were isolated by using the TRIzol® Reagent (Life technologies, Carlsbad, CA), and then the amount and quality of RNAs were assessed using the NanoDrop® ND-1000 Spectrophotometer (ThermoFisher, Waltham, MA), Bioanalyzer 2100, RNA 6000 Nano Kit and Small RNA Chip Kit (Agilent Technology, Santa Clara, CA). For miRNA library construction and sequencing, miRNAs were enriched using the PureLink™ miRNA Isolation Kit (Invitrogen, Carlsbad, CA), and then adaptors were hybridized and ligated to the 5′ and 3′ ends of these RNAs using the SOLiD™ Total RNA-Seq Kit (Applied Biosystems Inc., ABI, Carlsbad, CA). The ligated RNAs were used as templates for cDNA synthesis followed by cDNAs purification using the MinElute® PCR Purification Kit (QIAGENE, Valencia, CA). The cDNAs at 120–130 base pair (bp) were selected (Invitrogen, Novex® pre-cast gel products), amplified (ABI, SOLiD™ Total RNA-Seq Kit), purified (Invitrogen, PureLink™ PCR Micro Kit), and proceeded with SOLiD™ System template bead preparation (ABI, SOLiD™ RNA Barcoding Kit). The libraries were sequenced with a read length of 35 bp using the SOLiD 5500 xl sequencer.

### cDNA library construction and sequencing

RNA samples were collected as described for cDNA library construction. RNAs of desired time points were isolated as described and used RiboMinus™ (Invitrogen) to remove ribosomal RNAs. For the preparation of cDNA libraries, the RNAs were fragmented using the RNAase III by using the Library Builder™ Whole Transcriptome Core Kit (ABI) and cleaned up by using the Fragmented RNA Concentrator Module Kit (ABI). To amplify and purify the cDNA libraries we used the Library Builder™ Whole Transcriptome Core Kit (ABI). The cDNA libraries with a size between 200 and 300 bp were proceeded with the SOLiD™ System template bead preparation using the EZ Bead™ System (ABI). The libraries were sequenced with read length of 75 bp using the SOLiD 5500 xl sequencer.

### Identification of differential expressed genes and miRNAs

Raw reads from the SOLiD 5500 xl sequencing were analyzed using the LifeScope™ Genomic Analysis Software. Those reads were filtered for high quality, adaptor self-ligation, transfer RNAs and ribosomal RNAs (http://rfam.sanger.ac.uk/). These filtering steps resulted in high quality filtered reads representing zebrafish RNA sequences. The filtered reads were mapped to the zebrafish genome sequences (Zv9, http://www.sanger.ac.uk/resources/downloads/zebrafish/) and annotated either the miRBase v18 (http://www.mirbase.org/) for miRNAs or the zebrafish genome annotation file (Zv9/danRer7 from UCSC) for transcripts. Annotated reads were further normalized to the reads per kilo base per million (RPKM). The differentially expressed miRNA and genes were identified and compared using the Partek Genomics Suite (http://www.partek.com/).

### Quantitative PCR analysis

The total RNAs were isolated as described and genomic DNAs were removed by using the DNA-Free kit (Life technologies). One microgram of total RNAs was reversely transcribed to miRNAs and cDNAs by the miScript II RT Kit (QIAGENE). The quantitative polymerase chain reaction (qPCR) was performed using the miScript SYBR Green PCR Kit (QIAGENE) for miRNA quantification. The zebrafish U6 (5′-ACTAAAATTGGAACGATACAGAGA-3′) and *ef1α* served as the internal control for miRNA and mRNA, respectively. qPCR analyses were performed by using the iQ™ SYBR® Green Supermix in a CFX96 station (Bio-Rad, Hercules, California). Primers used are listed in the Additional file 1.

### Gene cloning

Coding sequences of *period 2* (*per2*, ENSDARG00000034503), *aryl hydrocarbon receptor nuclear translocator-like 1a* (*arntl1a*, ENSDARG00000006791), *basic helix-loop-helix family, member e41* (*bhlhe41*, ENSDARG00000041691) were amplified from the zebrafish total cDNAs by reverse transcriptase PCR (RT-PCR). Total RNAs of zebrafish embryos were isolated as described and reversely transcribed by the MMLV reverse transcriptase (Promega, Fitchburg, Wisconsin). Primers were designed according to the respective reference RNA sequence. The amplicons were sub-cloned into a pT2MUAS vector for Tol2-mediated transgenesis [35]. The vector also contains a viral 2A peptide sequence for the separation of the expressed protein from a nuclear reporter H2AmCherry used for founder fish screening.

### Ectopic expression and analysis of circadian clock genes in zebrafish larvae

One-cell stage zebrafish embryos were injected with 25 pg Gal4 mRNAs, 25 pg Toll2 mRNA and 50 pg plasmids and incubated under a 14 h/10 h dark/light cycle until examination. Embryos with H2AmCherry expression were selected for further analysis.

### Swimming behavior analysis

Five days post fertilization (dpf) larvae expressing with or without *per2*, *arntl1a* or *bhlhe41* were individually distributed into each well of a 24-well plate with 1.5 ml of 0.3× Danieau’s buffer and incubated for 4 h at 28.5 or 18 °C. Following incubation, the larvae were transferred to 28.5 °C and videotaped in a controlled light-on DanioVision observation chamber for 30 min. The tracking of swimming for each larva was analyzed during 0–10 min, 10–20 mins and 20–30 mins using the EthoVision XT 8.5 software (Noldus Information Technology, Wageningen, The Netherlands).

### Measurement of glucose concentration

Control or transient expressing embryos were cultured and cold shocked as described. Ten larvae for each treatment were collected at 0, 2, and 4 h after cold-shock, lysed in 200 μl RIPA buffer (ThermoFisher Scientific, Waltham, MA), and the glucose concentration was measured by using the Amplite^TM^ Fluorimetric Glucose Quantitation Kit (AAT-bioquest, Sunnyvale, CA).

### Reporter assay of *dre-mir-29b*

The 3′ untranslated region (UTR) of *per2* containing *dre-mir-29b* target site was amplified from the cDNA library by the forward (F) and reverse (R) primers of the following sequences. F: Bgll2-Stop-per2 ATCGAGATCTTAAAATTCCTTTCGCATTCACAA and R: SacI-per2-ATCGGAGCTCAGTCTGTGAGATCAGTTAAACCA. The amplicons were cloned into the pEGFP-C1 vector by selected restriction enzyme site SacI and Bgll2. Plasmids were mixed with or without *dre-mir-29b* morpholino (5′ACACTGATTTCAAATGGTGCTAGAT3′). The plasmid mix was injected into 1-cell zebrafish embryos and observed under epifluorescent microscopy at 10 hpf.

### Statistical analysis

All experimental values are presented as mean ± standard error and were analyzed by unpaired-sample Student’s *t*-test and one-way ANOVA.

## Results

### Cold-induced responses in zebrafish larvae

To investigate cold-induced responses in zebrafish, 96 hpf larvae were exposed to decreasing temperatures to monitor their mobility and mortality for 10 h (Table 1). Fish were mostly normal from 28.5 to 20 °C except for a few inactive fish at 20 °C. In contrast, most fish became motionless after longer incubation at 18 °C. Fish were inactive at 16 °C and were unresponsive upon touching. Death appeared at 12 °C and below. The mortality increased with lower temperatures and longer incubation (data not shown). Thus, we used 18 °C to study cold shock responses to avoid mortality and undesired health problems.

**Table 1:** Cold impairs swimming and causes mortality in zebrafish larvae

Incubation temperature	Hours Post Treatment				
°C	2	4	6	8	10
28.5	Normal	Normal	Normal	Normal	Normal
24	Normal	Normal	Normal	Normal	Normal
20	Normal	Normal	mostly normal but few inactive larvae	Mostly normal but few inactive larvae	Mostly normal but few inactive larvae
18 (% of inactive fish)	83	95	96	100	100
16	All fish were inactive and showed unbalance moves when disturbed				
12 (% of motility)	0	0	0	7.2	12

Four - day - old zebrafish larvae were incubated at decreasing temperatures from 28.5 to 12 °C. The mobility and viability were recorded every 2 h until 10 h post treatment

**Fig. 1:**
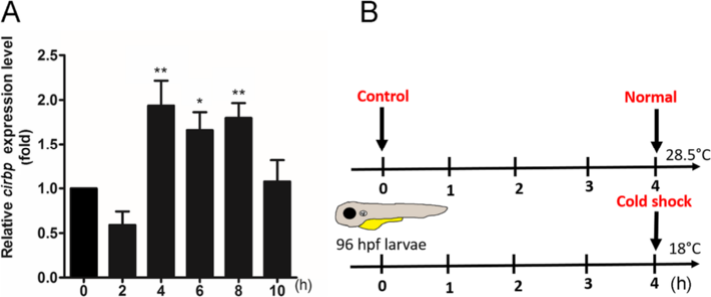
Cold shock treatment in zebrafish larvae. **a** Relative expression levels of *cirbp* gene in zebrafish larvae at 96 h post fertilization (hpf) were determined by q PCR at different time point post transfer from 28.5 to 18 °C. * and ** indicates *p* ≤ 0.05 and 0.01, respectively, comparing to the control at 0 h post transfer. **b** Scheme of cold exposure. Embryos were incubated at 28.5 °C until 96 hpf then transferred to 18 or 28.5 °C for an additional 4 h. Larvae before (control) and after treatments (normal and cold shock) were then collected for RNA-seq and miRNA-seq analyses as described in the Methods

To confirm that the exposure to 18 °C is able to elicit cold responses at the transcriptional level, we examined the change in gene expression of cold - inducible RNA binding protein (*cirbp*), a well-known cold inducible gene [11]. qPCR analysis showed that the *cirbp* expression was initially decreased at 2 h post induction (hpi), but significantly bounced back to about 2 folds at 4 hpi and then declined to normal at 10 hpi (Fig. 1a). It appeared that a 4-h incubation at 18 °C is sufficient to induce cold-dependent transcriptomic changes in zebrafish larvae.

qPCR analyses detected no notable change in expression of *cirbp* in samples before and immediately after transferring to 18 °C (data not shown). So the only time 0 control used was samples before cold shock. Larvae were then incubated at 28.5 °C (normal) or 18 °C (cold shock) and samples were taken from both conditions at 4 hpi (Fig. 1b). These experiments were repeated twice and a total of 6 different samples were subjected to small RNA and RNA NGS.

### Cold-induced miRNA sequencing profiles

**Fig. 2:**
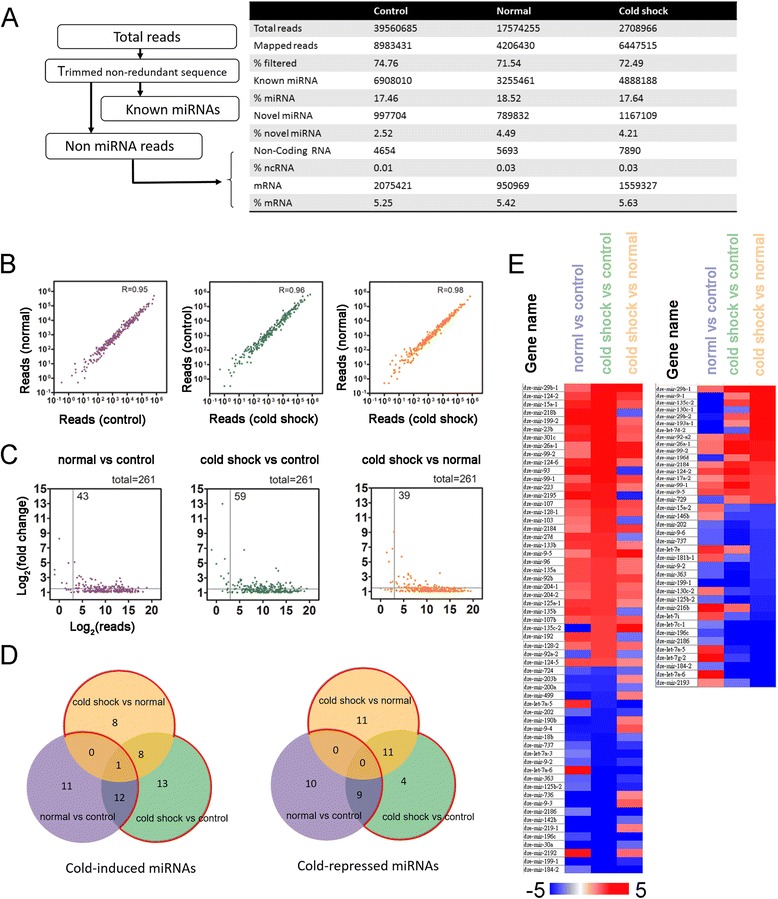
Analysis of cold shock - affected miRNAs by small RNA-seq in zebrafish larvae. **a** A pipeline is presented for small RNA identification at each filtration step (*left*). The number of reads and percentage passed each filtration step of each treatment are shown on the *right chart*. **b** Scatter plots show comparisons of RPKM between treatments. Log plots are shown in (**c**) for those cold-affected genes with a change in expression ≥2 folds. The *vertical line* in each graph indicates where log2 (RPKM) equal to 1.07. Only *dots* with values greater than 1.07 were subjected to further analyses. **d** The numbers of cold shock-affected (≥1.5 folds changes) miRNA are presented in Venn diagram as shown in Fig. 1e. **e** Heat maps profiles for change in expression of selected miRNAs

To clarify the roles of miRNA on transcriptomic plasticity in cold responses, we performed both small RNA-seq and RNA-seq analyses in zebrafish larvae. Six RNA samples were extracted from three groups (control, normal and cold shock) as previously described, enriched for 18–30 bp small RNAs for the cDNA library construction and subjected to NGS using the SOLiD 5500 xl. A total of 39,560,685; 17,574,255, and 27,708,966 sequence reads from the first batch were obtained from the control, normal and cold shock sample, respectively (Fig. 2a, the total reads of the second batch were listed in Additional file 2: Figure S2A). The sequencing data was analyzed using the LifeScope™ Genomic Analysis Software and normalized using the Partek Genomics Suite. The length distribution of each library reads were ranged between 18 and 30 bp, and the major sizes peaked around 21~24 bp that matched the general size distribution of miRNAs (Additional file 2: Figure S1A, S2B). Abundant reads were located at chromosome 4, 10, 20, and 25 in different libraries (Additional file 2: Figure S1B, S2C). After filtering out short and adaptor-contaminated reads, about 17–19% miRNAs were identified. The reads and the percentage of different RNA components (miRNA, noncoding RNA, mRNA, novel RNA) were similar among samples (Fig. 2a). To examine the overall effects of cold shock on miRNAtome, the batches of data were normalized by reads count and subjected to principle component analysis to perform the quality check of samples as shown in Additional file 2: Figure S3. Then we mapped the sequencing reads to the miRBase v18, which contains 344 zebrafish miRNAs and found 261 known miRNAs. After normalizing two batches of data from three groups, the reads from different treatments were highly correlated (Fig. 2b). It suggests that the majority of miRNAs remained relatively constant during development and cold shock. Figure 2c shows the log plots of the fold of change in miRNA expression between treatments. We considered the fold of change ≥1.5 folds to be significant. Transcriptomic profiles are changing rapidly during embryonic development. The altered miRNAs between control and normal were thought to be development-dependent. After taking out development-dependent miRNAs, we identified 29 cold-induced miRNAs and 26 cold-repressed miRNAs (within the red boundary in Fig. 2d). The heat map of miRNAs with a change ≥1.5 folds are shown in Fig. 2e and Additional file 3. The top 5 mRNAs affected are listed in Table 2 and the list of affected - miRNAs can be found in Additional file 4.

**Table 2:** Cold shock-induced differential expression of known miRNAs

miRNA	Mature miRNA sequence	Fold change
Up-regulated miRNAs		
Dre-mir-29b-1	UAGCACCAUUUGAAAUCAGUGU	3.90
Dre-mir-99-2	AACCCGUAGAUCCGAUCUUGUG	2.04
Dre-mir-99-1	AACCCGUAGAUCCGAUCUUGUG	1.79
Dre-mir-92a-2	UAUUGCACUUGUCCCGGCCUGU	1.75
Dre-mir-2184	AACAGUAAGAGUUUAUGUGCU	1.73
Down-regulated miRNAs		
Dre-mir-737	AAUCAAAACCUAAAGAAAAUA	−1.71
Dre-mir-9-2	UCUUUGGUUAUCUAGCUGUAUGA	−1.75
Dre-mir-363	AAUUGCACGGUAUCCAUCUGUA	−1.79
Dre-mir-125b-2	UCCCUGAGACCCUAACUUGUGA	−1.91
Dre-mir-199-1	CCCAGUGUUCAGACUACCUGUUC	−2.79

After eliminating miRNAs whose expression might be changed due to development process during incubation (control vs normal). The top 5 of 29 up- and 26 down-regulated known miRNAs (annotated in miRBase v18) are shown, along with their sequences and fold changes compared to the control groups

### Validation of miRNA-seq data with qPCR

**Fig. 3:**
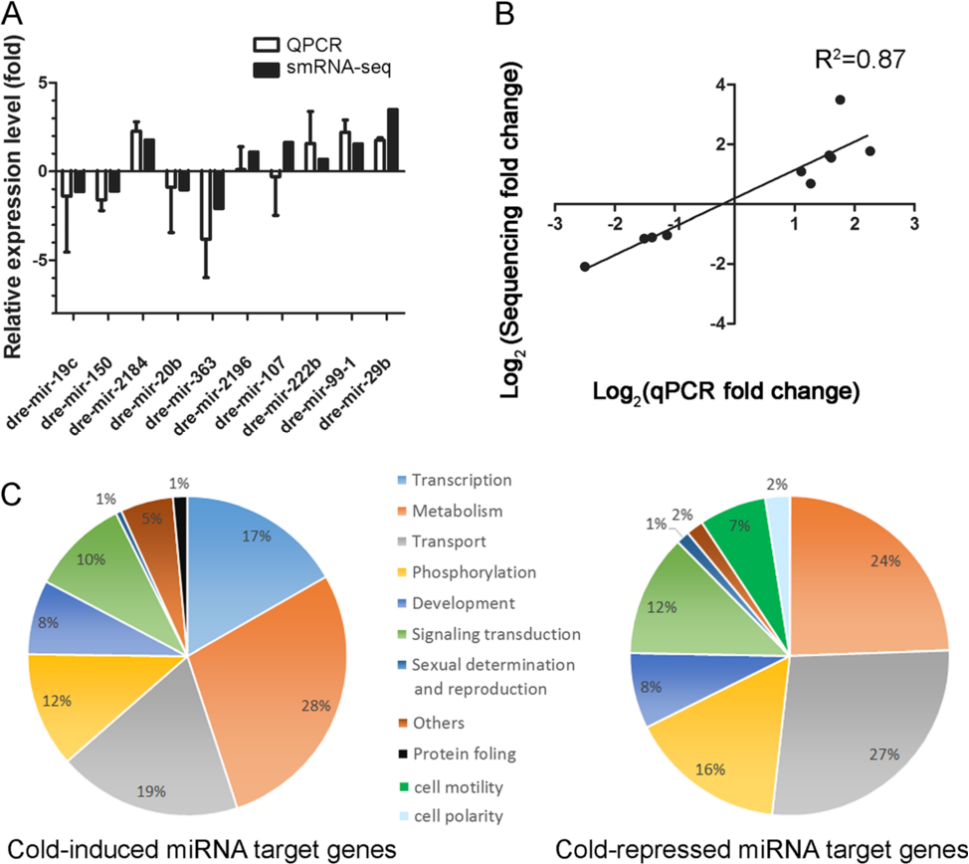
Validation of small RNA-seq data and biological process pathway summary for the target genes of significantly affected miRNA upon cold shock. **a** Comparison of change in relative expression of selected miRNAs determined by NGS and qPCR (*N* = 3) (**b**) Pearson’s correlation between small RNA-seq and qPCR data (correlation coefficient (R^2^ = 0.807). **c** Pie charts show the proportions of cold-induced (*left*) and repressed (*right*) miRNAs whose target genes are involved in different biological processes with a *p* value ≤ 0.05

We performed qPCR analysis to validate the changes of the cold-affected miRNAs (*dre-mir-29b*, the most up-regulated miRNA, and 9 other randomly selected miRNAs) and compared them to those observed in small RNA-seq results (Fig. 3a). The Pearson’s correlation coefficient between two analyses is 0.87. This is relatively high and suggests the reliability of small RNA-seq results (Fig. 3b).

### Function annotation for target genes of the cold-dependent miRNAs

The cold-dependent target genes of each miRNA were predicted by TargetScanFish release 6.2 (http://www.targetscan.org/fish_62/) with the total context+ score −0.3 as a cutoff [36]. We obtained 6263 and 5381 targets of cold-induced and -repressed miRNAs, respectively (Additional file 4). We then conducted the target genes functional annotation using The Database for Annotation, Visualization and Integrated Discovery (DAVID) Bioinformatics Resources 6.7 (http://david.abcc.ncifcrf.gov/) [37]. The top 10 biological processes were ranked according to *P* value (Table 3 and Additional file 5). The *P* value is an indication of gene enrichment in annotated biological process. The percentages of affected miRNA target genes with a value ≤ 0.005 are illustrated in pie graphs (Fig. 3c). In general, both up- and down-regulated miRNA target genes were enriched in processes like metabolism, transport, phosphorylation, development, signaling transduction, sexual determination and reproduction. In contrast, transcription-related genes were only enriched in the up-regulated miRNA target genes, but cell movement and motility-related genes were abundant in the down-regulated miRNA target genes.

**Table 3:** The top 10 biological processes for cold-related miRNA target genes

Biological Processes of	*P* value*
Cold-induced miRNA target genes	
Regulation of transcription, DNA-dependent	3.30E-04
Polyol metabolic process	3.40E-04
Regulation of RNA metabolic process	5.20E-04
Regulation of transcription	6.70E-04
Regulation of small GTPase mediated signal transduction	1.10E-03
Ion transport	1.10E-03
Glycerol metabolic process	1.30E-03
Alditol metabolic process	1.30E-03
Organophosphate metabolic process	1.50E-03
Metal ion transport	1.60E-03
Cold-repressed miRNA target genes	
Metal ion transport	1.60E-04
Protein amino acid phosphorylation	3.60E-04
Intracellular signaling cascade	7.60E-04
Ion transport	8.50E-04
Cation transport	1.80E-03
Potassium ion transport	1.80E-03
Regulation of small GTPase mediated signal transduction	1.80E-03
Organophosphate metabolic process	3.10E-03
Monovalent inorganic cation transport	3.60E-03
Regulation of Ras protein signal transduction	4.70E-03

*calculated by Fisher Exact which is adopted to measure the gene-enrichment in annotation biological process

Target genes of 29 up- and 26 down-regulated known miRNAs were predicted by TargetScan (Additional file 5). The target genes were annotated by DAVID bioinformatics resources 6.7. The top 10 biological processes of the target genes and *P* values are shown

We also predicted the top 10 cold-induced and cold-repressed miRNA-mediated signaling pathways by using the Kyoto Encyclopedia of Genes and Genomes (KEGG) pathway database (Table 4 and Additional file 5). In the up-regulated group, most genes were involved in metabolism, including cysteine and methionine metabolism, fructose and mannose metabolism, glycerophospholipid metabolism, inositol phosphate metabolism and sphingolipid metabolism. One pathway was related to extracellular matrix-receptor interaction and the other one was related to biosynthesis of unsaturated fatty acids. In the cold-repressed group, many target genes were involved in biosynthesis and metabolism pathways, including chondroitin sulfate biosynthesis, synthesis and degradation of ketone bodies, O-Glycan biosynthesis, glycosphingolipid biosynthesis, biosynthesis of unsaturated fatty acids, sphingolipid metabolism and alanine, aspartate and glutamate metabolism. Some cold-repressed miRNA target genes were also found in the phosphatidylinositol, notch and lysozyme signaling pathways.

**Table 4:** The top 10 KEGG pathways for cold-related miRNA target genes

KEGG pathway of	*P* value*
Cold-induced miRNA target genes	
Cysteine and methionine metabolism	0.0027
ECM-receptor interaction	0.0035
Fructose and mannose metabolism	0.005
Biosynthesis of unsaturated fatty acids	0.011
Glycerophospholipid metabolism	0.011
Inositol phosphate metabolism	0.014
Sphingolipid metabolism	0.022
Melanogenesis	0.028
GnRH signaling pathway	0.029
Circadian rhythm	0.034
Cold-repressed miRNA target genes	
Chondroitin sulfate biosynthesis	0.00081
Synthesis and degradation of ketone bodies	0.0092
Phosphatidylinositol signaling system	0.017
Sphingolipid metabolism	0.019
Notch signaling pathway	0.023
O-Glycan biosynthesis	0.025
Lysosome	0.03
Alanine, aspartate and glutamate metabolism	0.035
Glycosphingolipid biosynthesis	0.036
Biosynthesis of unsaturated fatty acids	0.038

*calculated by Fisher Exact which is adopted to measure the gene-enrichment in annotation KEGG pathway

Target genes of 29 up- and 26 down-regulated known miRNAs were predicted by TargetScan (Additional file 5). The targets genes were annotated by DAVID bioinformatics resources 6.7. The top 10 KEGG pathway of the target genes and *P* values are shown

### Cold-induced RNA sequencing profile

**Fig. 4:**
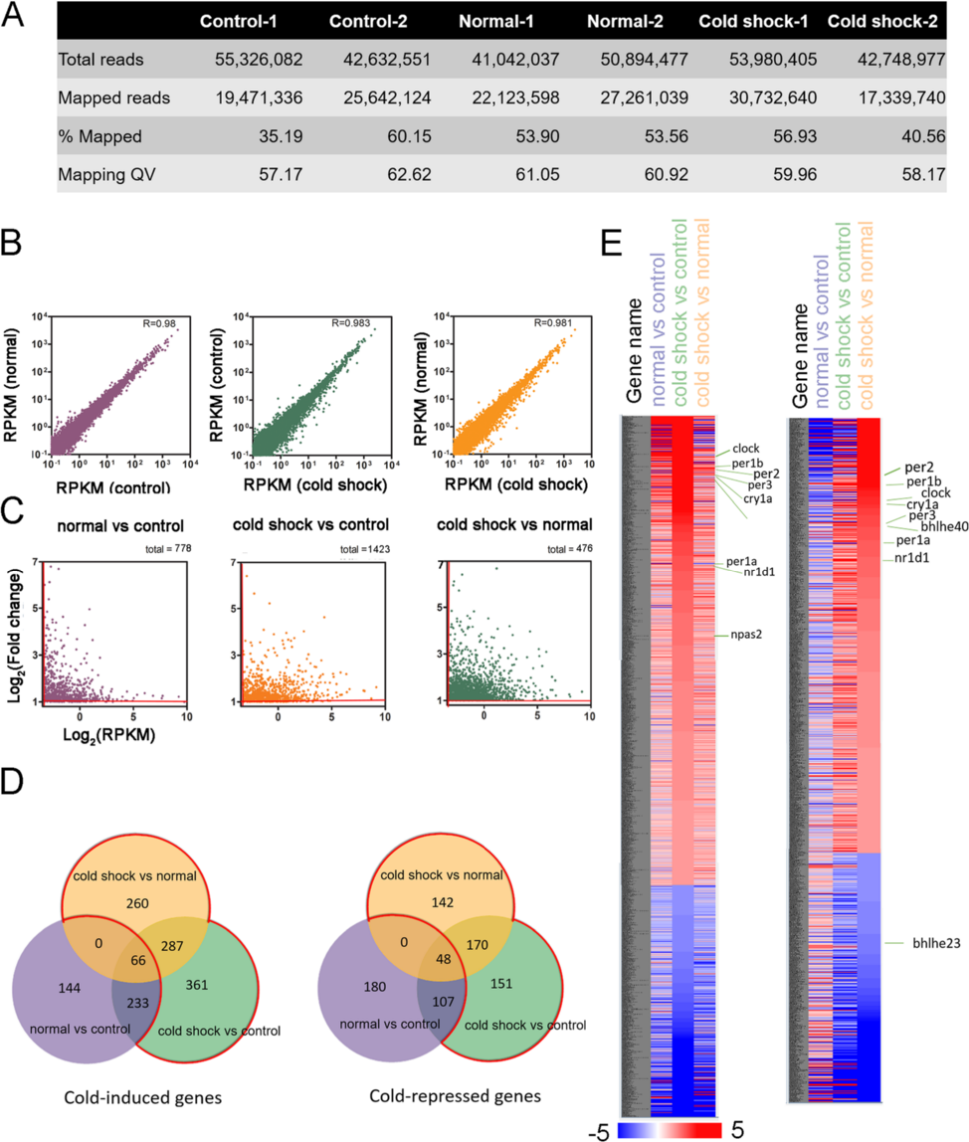
Transcriptomic analysis of cold shock-induced genes in zebrafish larvae. **a** The number of reads and percentage passed each filtration step of each treatment are shown on the *right chart*. **b** Scatter plots show comparisons of reads per kilo base per million (RPKM) between treatments. Log plots are shown in (**c**) for those cold-affected genes with a change in expression ≥ 2 folds and RPKM ≥ 0.1. The total dot numbers are shown on the *right top corner* of each graph. **d** Venn diagrams show the numbers of cold shock-induced and repressed genes between treatments. **e** Heat maps profiles for mRNA expression of selected genes with annotations of circadian - related genes

To validate our predicted miRNA targets, six RNA samples were extracted from zebrafish larvae, treated and processed as described previously to perform RNA-seq analysis using the SOLiD 5500 xl. The transcriptomic differences between three groups of fish were similarly analyzed as described in the miRNAtome analysis. Total numbers of reads and mapped reads of duplicates from three different treatments are shown in Fig. 4a. After normalizing two batches of data from three groups, the RPKM from different treatments (Fig. 4b) were highly correlated. It suggests that the majority of mRNAs remained relatively constant during development and cold shock. Figure 4c shows the log plots of the fold of change in RNA expression between treatments. We set the fold change ≥ 2 folds and RPKM ≥ 0.1 to be significant. After taking out development-dependent RNAs, we obtained 908 cold-induced genes and 463 cold-repressed genes (within the red boundary in Fig. 4d). The list of affected genes can be found in Additional file 6. The heat map of genes with a change ≥ 2 folds are shown in Fig. 4e and Additional file 3. Among them, the genes related to circadian rhythm are indicated.

### Validation of the cold-induced changes in circadian clock genes with qPCR

The circadian clock genes were also shown to be affected during cold acclimation in zebrafish [38]. However, their roles in the cold-induced response have not been elucidated. We first performed qPCR analysis to validate the changes of those circadian clock genes, which include *per2*, *per1a*, *clock3*, *clock*, *arntl1a*, *arntl1b*, *nr1d*, *cry1a*, and *bhlhe41*, under cold shock or normal conditions. The qPCR data was compared to those results by the RNA-seq (Fig. 4a)*.* The Pearson’s correlation coefficient between two analyses is 0.807 (Fig. 4b) that is relatively high and suggests the reliability of RNA-seq results.

### Function annotation of cold-dependent genes in transcriptome

**Fig. 5:**
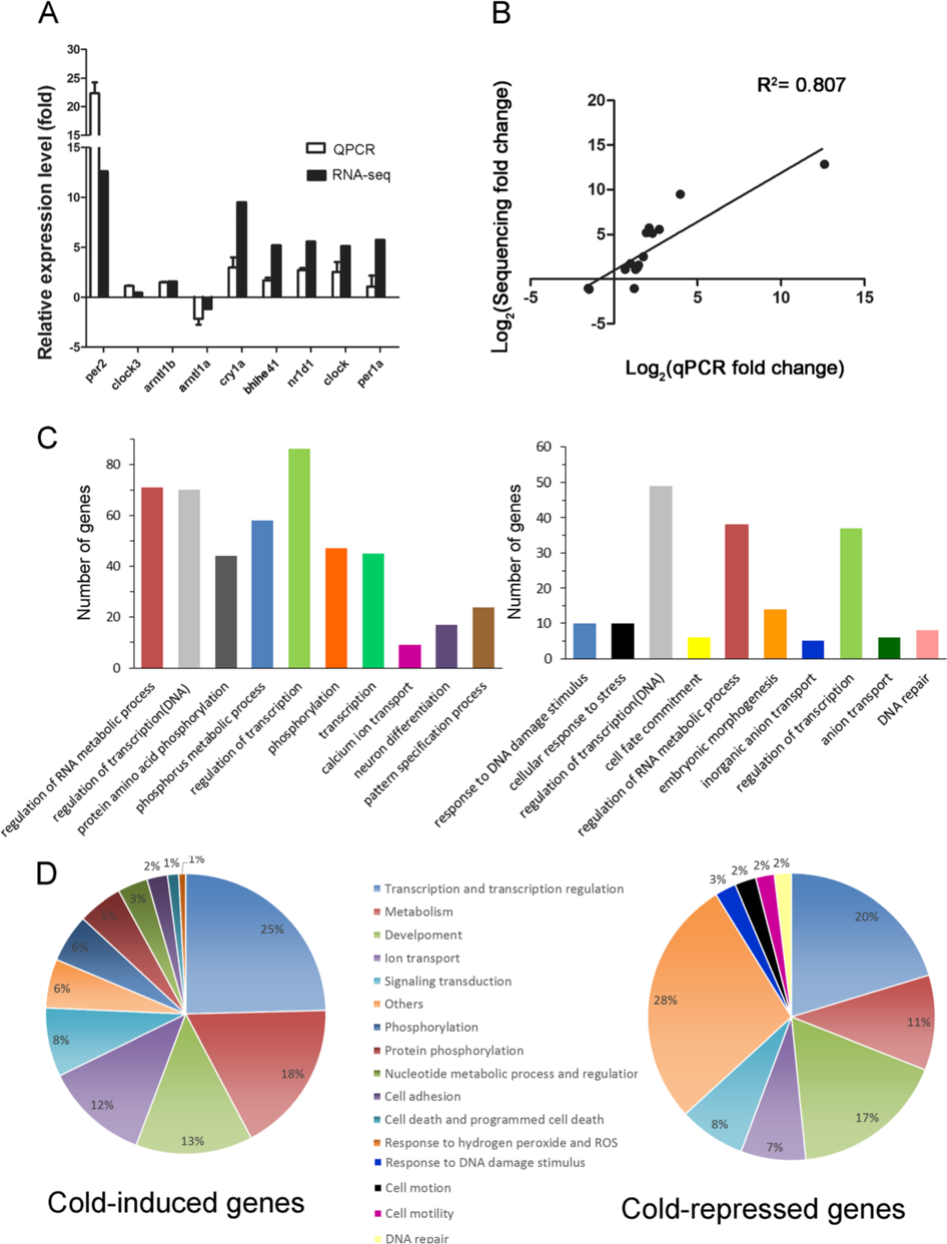
Validation of RNA-seq data and biological process pathways summary of the genes significantly changed upon cold shock. **a** Comparison of change in relative expression of circadian clock genes determined by NGS and qPCR (*N* = 3). **b** Pearson’s correlation result between RNA-seq and qPCR data. (Correlation coefficient = 0.807) (**c**) Numbers of cold-induced (*left*) and repressed (*right*) genes belong to the top 10 biological processes. **d** Pie charts show the proportions of cold-induced (*left*) and repressed (*right*) genes which involve in different biological processes with a *p* value ≤ 0.05

To reveal the biological interpretation of cold-dependent genes, we conducted the genes’ functional annotation using DAVID Bioinformatics Resources 6.7 (http://david.abcc.ncifcrf.gov/) to find the enriched biological processes by the Gene Ontology (GO) term analysis. The *P* value ranking indicates gene enrichment in annotated biological processes. The top 10 biological processes, such as regulation of RNA metabolic process, are listed in Table 5 and the number of genes belonging to each process are shown in Fig. 5c. The percentages of affected genes with a value ≤ 0.1 are illustrated in a pie graph (Fig. 5d). In general, both up- and down-regulated genes were enriched in regulation of RNA metabolic processes, regulation of transcription*,* DNA-dependent and regulation of transcription. In contrast, protein amino acid phosphorylation, phosphate metabolic process, calcium ion transport, neuron differentiation and pattern specification process were only enriched in the up-regulated genes. Response to DNA damage stimulus, cellular response to stress, cell fate commitment, embryonic morphogenesis, inorganic anion transport, anion transport and DNA repair were abundant in the down-regulated genes.

**Table 5:** The top 10 biological processes for cold-related genes

Biological Processes of	*P* value*
Cold-induced genes	
Regulation of RNA metabolic process	1.50E-03
Regulation of transcription, DNA-dependent	1.80E-03
Protein amino acid phosphorylation	1.90E-03
Phosphate metabolic process	2.10E-03
Rgulation of transcription	5.40E-03
Phosphorylation	6.80E-03
Transcription	9.50E-03
Calcium ion transport	0.01
Neuron differentiation	0.015
Pattern specification process	0.019
Cold-repressed genes	
Response to DNA damage stimulus	0.01
Cellular response to stress	0.018
Regulation of transcription	0.019
Cell fate commitment	0.027
Regulation of RNA metabolic process	0.03
Embryonic morphogenesis	0.033
Inorganic anion transport	0.037
Regulation of transcription, DNA-dependent	0.04
Anion transport	0.04
DNA repair	0.047

*calculated by Fisher Exact which is adopted to measure the gene-enrichment in annotation biological process

The cold-related genes (Additional file 6) were annotated by DAVID bioinformatics resources 6.7. The top 10 biological processes of the cold-related genes and *P* values are shown

We also analyzed the cold-affected genes in the KEGG pathway database (Table 6) [39]. In the up-regulated groups, most of the genes were involved in MAPK, hedgehog, GnRH and insulin signaling pathways. Other genes were enriched in immunity-related pathways like retinoic acid-inducible gene 1 (RIG-I)-like receptor and oligomerization domain (NOD)-like receptor, circadian rhythm, melanogenesis and adhesion molecule. In the cold-repressed genes, only neuroactive ligand-receptor interaction and steroid biosynthesis were significantly enriched.

**Table 6:** The top 10 KEGG pathways for cold-related genes

KEGG pathway of	*P* value*
Cold-induced genes	
MAPK signaling pathway	2.60E-05
Circadian rhythm	2.60E-04
RIG-I-like receptor signaling pathway	0.011
GnRH signaling pathway	0.025
Cell adhesion molecules (CAMs)	0.026
Adherens junction	0.034
Melanogenesis	0.036
Hedgehog signaling pathway	0.049
NOD-like receptor signaling pathway	0.056
Insulin signaling pathway	0.057
Cold-repressed genes	
Neuroactive ligand-receptor interaction	3.90E-03
Steroid biosynthesis	6.50E-03

*calculated by Fisher Exact which is adopted to measure the gene-enrichment in annotation KEGG pathway

The cold-related genes (Additional file 6) were annotated by DAVID bioinformatics resources 6.7. The top 10 KEGG pathway of the genes and *P* values are shown

### Correlation of expression profiles of miRNA and mRNA

To determine the correlation between cold-affected miRNAtome and transcriptome, we further analyzed the expression of cold-affected miRNAs and their target genes as shown in Additional file 7. The affected miRNAs should be negatively correlated with their target mRNAs. However, it is not always the case. Only 23.8% of up-regulated miRNAs resulted in down-regulation of target mRNAs. On the other hand, 75.4% of down-regulated miRNAs caused up-regulation of target mRNAs. It appears that the release of miRNA inhibition may have a bigger impact on the expression of target mRNAs.

### Overexpression of *per2* assists in cold recovery in zebrafish larvae

**Fig. 6:**
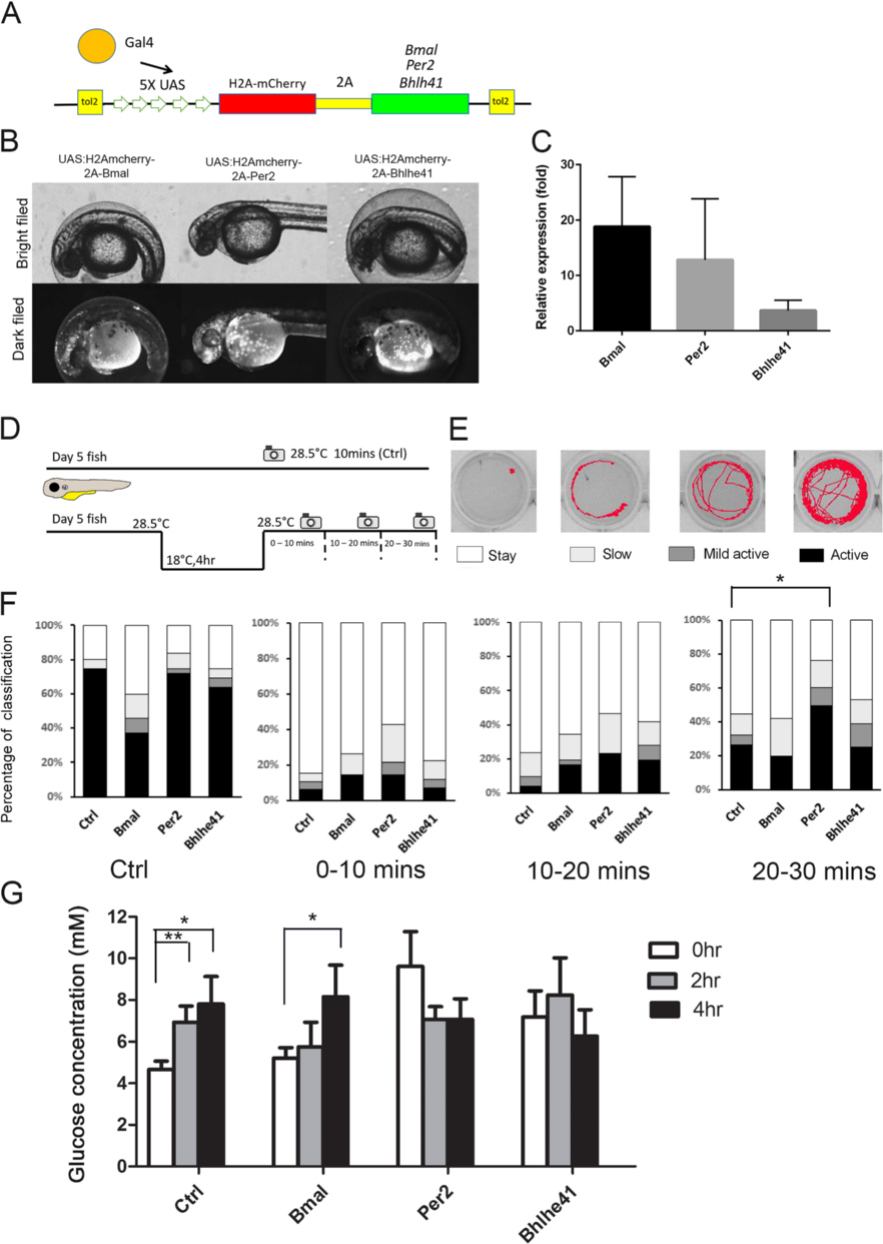
Overexpression of core clock gene *per2* or *bhlhe41* increases cold tolerance. **a** The design of the Gal-UAS driven circadian clock gene expression construct is shown. H2AmCherry (*red*) is placed upstream of clock gene (*green*) that will be cleaved by a 2A peptide (*yellow*). **b** 1-Cell embryos were injected with indicated plasmids and examined under bright (*upper row*) and dark filed (*bottom row* through a rhodamine filter cube). Mosaic expression of mcherry indicates the expression of *bmal*, *per2*, or *bhlhe41* in 48 hpf embryos. **c** Normalized expression level of clock genes compared to control embryos at 5 days post fertilization by qPCR (*N* = 3). **d** Scheme of cold recovery assay at 5 days post fertilization fish. **e** Classifications of swimming track recorded for 10 mins. **f** Quantitative analysis of swimming patterns for zebrafish larva transient expressed without (control) and with designated circadian clock gene at different time periods after recording. **g** Glucose concentration were measured in 4 dpf zebrafish larval lysate collected at different time point after cold shock. *P ≤ 0.05, **p ≤ 0.01

Biological processes of the Gene Ontology (GO) term analysis shows that circadian rhythm is enriched at the first category - regulation of RNA metabolic process as shown in Additional file 8. As described previously, the roles of circadian clock genes in the cold shock responses are still illusive. In the molecular level, the core clock genes, CLOCK and NPAS2/BMAL1, induce expression of repressors, PER1-3/CRY1-2 to form an interacting feedback loop to sustain the daily oscillation [40]. BHLHE41 plays a relative minor role that functionally resembles the inhibitor of the core clock gene complex and oscillates similarly with the PER1-3/CRY1-2 complex [41]. It has been reported that the dynamics of circadian clock genes are highly correlated with cell metabolism [42, 43]. We hypothesized that the circadian gene-dependent changes in cell metabolism may help in cold resistance. To test this hypothesis, we selected three circadian clock genes, *arntl1a (bmal)*, *bhlhe41* and *per2*, which had different levels of change (1.14, 2.4 and 12.6 folds, respectively) upon cold shock, to perform functional assays. We designed overexpressing plasmids containing Tol2, 5XUSA element, different circadian clock gene coding region sequence and H2AmCherry indicator. The circadian clock gene and mCherry were intervened with a 2A peptide sequence for separation upon translation. Different plasmids were separately injected into 1-cell embryos with *gal4* mRNAs (Fig. 6a). The ectopic expression of these plasmids were screened by the H2AmCherry fluorescence under epifluorescent microscope as an indirect indication of circadian clock gene overexpression (Fig. 6b). To validate this, we performed qPCR analyses and found that the expressions of *bmal*, *per2* and *bhlhe41* were increased to 18.86 ± 9.1, 12.85 ± 11.17 and 5.02 *±* 1.88 folds, respectively (Fig. 6c, *N* = 3). These fish with transient expression of circadian clock genes were then used for the cold tolerance assay.

Zebrafish swimming behavior has been used as an indicator for cold tolerance [44]. We first cultured the circadian clock gene overexpressing fish under a 14 h/10 h dark light cycle for 5 days and incubated in 18 °C for 4 h. We then transferred fish to a 28.5 °C observation chamber to record the spontaneous swimming behavior from 0 to 30 mins after transfer (Fig. 6d). The moving paths at different intervals after cold recovery (0–10, 10–20 and 20–30 min) were recorded and analyzed according to the intensities of four classes of swimming activity (Fig. 6e). The *per2* and *bhlhe41* overexpressing fish had higher swimming activity than those of control and *arntl1a* overexpressing fish at 10–20 mins interval (% of moving fish: Ctrl: 23 ± 7%, *n* = 40; Bmal: 35 ± 7%, *n* = 34; Per2: 39 ± 15%, *n* = 35, Bhlhe41: 42 ± 15%, *n* = 36). The *per2*-overexpressing fish exhibited the most active swimming behavior during the 20–30 min interval (% of moving fish: Ctrl: 44 ± 9.2%, *n* = 32; Bmal: 42 ± 12%, *n* = 36; Per2: 76 ± 12%, *n* = 30; Bhlhe41: 53 ± 10%, *n* = 34) as shown in Fig. 6f.

To examine cell metabolism, we measured the change in glucose concentration in the circadian clock gene overexpressing larvae. In control and *bmal* (*arntl1a*)-overexpressing fish, the glucose concentration was gradually increased during cold shock. In contrast, the *bhlhe41*- and *per2-*overexpressing fish had a higher basal glucose level and the level was not significantly changed during cold shock (Fig. 6g).

### *Dre-mir-29b* targets *per2* mRNA and regulates its expression dynamically during cold treatment

**Fig. 7:**
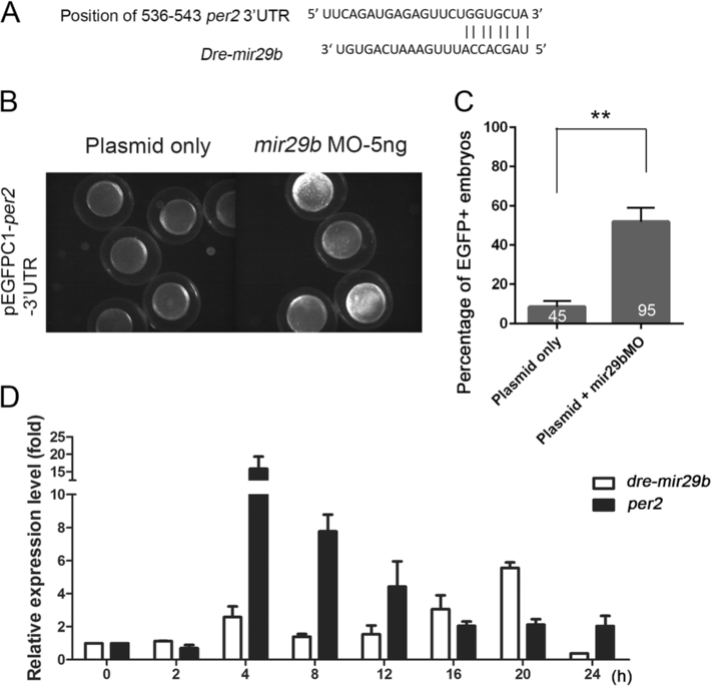
*Dre-mir-29b* may regulate *per2* expression during cold acclimation. **a** *p*er2 is a predicted target of *dre-mir-29b*. A fragment of 3′ untranslated region (3′UTR) containing the complementary nucleotides of *dre-mir-29b* is shown. **b** 1-cell embryos were injected 50 pg pEGFPC1 bearing 3′UTR of *per2* target site in the presence or absence of 5 ng *mir-29b* MO and examined at 90% epiboly stage under epifluorescent microscopy using a FITC cube. **c** The percentages of EGFP - positive embryos injected without or with *dre-mir-29b* are shown. The numbers of embryos used in each treatment are presented at the *bottom* of each column. (*N* = 3), **p ≤ 0.01. **d** Embryos were treated with cold shock, lysed at designated time post cold shock, and their RNAs were extracted for qPCR analysis for *dre-mir-29b* (*open bar*) and *per2* (*filled bar*)

The increase in cold tolerance in *per2*-overexpressing fish suggests a pivotal role of circadian clock genes against cold stress. Comparing the expression level of miRNA-seq and mRNA-seq of gene of interest, we identified several cold-dependent miRNAs that may regulate circadian clock genes (Tables 7 and 8). We selected *dre-miR-29b* to illuminate how miRNA affect transcriptome plasticity during cold shock (Fig. 7a). *Dre-miR-29b* is the top one cold-induced miRNA with a predicted target of *per2*. To address this, we injected pEGFPC1-*per2* 3′UTR with or without *dre-mir-29b* morpholino oligonucleotides (MO) (Fig. 7b). The co-injection of *mir-29b* MO significantly enhanced the % of embryos showing the fluorescence from the pEGFPC1-*per2* 3′UTR (51.9%, *n* = 95, *N* = 3) compared to that of plasmid only embryos (8.65%, *n* = 49, *N* = 3) as shown in Fig. 7c. This data suggests that *dre-mir-29b* MO can prohibit *dre*-*mir-29b* targeting *per2* mRNAs.

**Table 7:** Cold-affected circadian clock genes and associated miRNAs

Circadian rhythm	miRNA
Basic helix-loop-helix domain containing, class B, 3 like	Dre-mir-92, dre-mir-107, dre-mir-103
Cryptochrome 3 (*cry3*)	Dre-mir-29
Aryl hydrocarbon receptor nuclear translocator-like 1b (*arntl1b*)	Dre-mir-99
Clock homolog 3 (*clock3*)	Dre-mir-29, dre-mir-96, dre-mir-499
Period homolog 2 (*per2*)	Dre-mir-29,dre-mir-18
Period homolog 1a (*per1a*)	Dre-mir-135
Nuclear receptor subfamily 1 (*nr1d1*)	Dre-mir-204
Basic helix-loop-helix family, member e40 (*bhlhe40*)	Dre-mir-128, dre-mir-181
Casein kinase 1, epsilon	Dre-mir-130, dre-mir-301
Casein kinase 1, delta a	Dre-mir-29

**Table 8:** Cold-affected miRNAs and corresponding circadian clock genes

MicroRNA	Up/Down	Target
*Dre-mir-29b*	Up	*Per2, Per3, Cry3, Ck1,Clock3*
*Dre-mir-135*	Up	*Per1a, Nr1d4a*
*Dre-mir-99*	Up	*Arntl1b*
*Dre-mir-92a*	Up	*Bhlhe41*
*Dre-mir-96*	Up	*Clock3*
*Dre-mir-204*	Up	*Nr1d1*
*Dre-mir-128*	Up	*Bhlhe40*
*Dre-mir-107*	Up	*Bhlhe41*
*Dre-mir-2192*	Down	*Clock*
*Dre-mir-18*	Down	*Per2, Per3,Bhlhe41*
*Dre-mir-202*	Down	*Nr1d2b*
*Dre-mir-181*	Down	*Bhlhe40, Nr1d12a*
*Dre-mir-363*	Down	*Nr1d2a, Bhlhe41*
*Dre-mir-130*	Down	*Ck1ε, Nr1d12a*
*Dre-mir-301*	Down	*Ck1ε*
*Dre-mir-184*	Down	*Cry1b*
*Dre-mir-499*	Down	*Clock3, Nr1d14b*

Up/Down: Significantly up-regulated or down-regulated miRNAs are listed, the comprehensive Targeting clock genes are listed at the right column

To further study the dynamic expression of *per2* and *dre-mir-29b* during cold shock, we measured their expression levels at a shorter duration (every 2–4 h) until 24 hpi (Fig. 7d). It appeared that both *dre-mir-29b* and *per2* expression was elevated at 4 hpi. The *per2* expression declined afterward. In contrast, the *dre-mir-29b* expression increased afterwards, but a sudden drop was observed at 24 hpi. These results suggested that *dre-mir-29b* may act to control the *per2* expression-induced by cold shock.

## Discussion

Cold shock is a general, but harmful, environmental stress for ectothermic animals. We performed high throughput small RNA-seq and RNA-seq experiments to investigate potential regulatory mechanisms for cold-inducible responses and to characterize cold-affected miRNAs and genes in zebrafish larvae. We found 29 cold-induced miRNAs and 26 cold-repressed miRNAs in the microRNAtome analysis. We also discovered 908 cold-induced genes and 468 cold-repressed genes in the transcriptome analysis. Among them, circadian clock genes and others were enriched in both analyses. Thus, we overexpressed one of the cold-induced core clock gene *per2* and showed a notable increase in cold tolerance in zebrafish larvae. We further validated the targeting of *per2* by its associated miRNA, *dre-mir-29b*, which was also induced by cold shock. This suggests that miRNA can serve as a fine tuner for regulating the transcription of cold-inducible genes during cold stress.

An acute temperature drop may result in lethality in aquatic animals. Therefore, we first determined a permissible temperature (18 °C) that could induce adaptive cold responses without deleterious effects on fish. Cirbp, a cold shock-inducible protein, is served as a molecular indicator for cold shock. Cirbp acts as a RNA chaperone to unfold RNA secondary structures to facilitate translation. The elevation of *cirbp* expression has been known to be a universal marker upon cold exposure [1]. We measured the *cirbp* expression by qPCR and found that 4 h is the minimum duration which could cause significant gene induction. Furthermore, transcriptome and miRNAtome profiles change rapidly during the larvae stage, however, it was generally ignored in previous studies [15, 38]. Therefore, we ruled out the genes, which might be changed during development to be sure that the up- and down- regulated genes selected are truly cold shock induced.

The cold-affected genes and miRNAs are involved in many crucial biological processes. The most dominate pathways are attributed to transcription regulation, protein phosphorylation and transport. According to previous studies, both cold acclimation and cold shock could affect RNA transcription [11, 15, 45]. In our RNA-seq data, around 20% cold-regulated genes encode transcription factors such as *her, hsf2*, *hsf1*, *dmrt3a*, *pou1f1*, *atf3*, *gata1a*, *cebpb*, *fosl1, bhlhe41*, *bhlhe40*, *nr1d4b*, *nr1d4a*, *nr1d1* and *nr1d2a*. Among these genes, *bhlhe41*, *bhlhe40*, *nr1d4b*, *nr1d4a*, *nr1d1*, *nr1d2a* have been reported to be enriched in cold-induced carp larvae [11], suggesting transcription of certain genes should be induced by the cold in the fish larvae stage. The protein phosphorylation landscape is changed during cold treatment in aquatic creatures [46]. In the same token, we also observed the up-regulation of many kinase proteins such as MAP kinase interacting serine/threonine kinase 1 cascades, calcium/calmodulin-dependent protein kinase, protein kinase C and casein kinase 1. Furthermore, we found the changes in expression of multiple ion transporters (*atp2a2a, atp2a2b, atp2b3a, cacnb2a, cacna1c, ryr1b, and slc8a2b*). It implies that cold shock may inhibit ion pumps and channels that results in imbalance of osmoregulation in fish [47, 48]. Studies also revealed that cold can affect the oxidation reduction processes [15, 45]. However, these processes were not significantly changed in our analyses, which may reflect different cold treatment regime or animals used. In general, our results are in good agreement with previous reports.

Circadian clock genes, including *bhlhe40*, *npas2*, *nr1d1*, *nr1d4*, *per1a*, *per1b* and *per2*, are under the regulation of RNA metabolic process. The RNA metabolic process is the No. 3 and No. 1 in the *cold-induced miRNA target genes* (Table 3) and the *cold-induced genes* (Table 5), respectively. We analyzed genes belonging to the regulation of RNA metabolic process by using the KEGG pathway analysis and found that circadian rhythm is one of two most enriched pathways as shown in the second worksheet of Additional file 8. It gains further proof that circadian rhythm is listed as the No. 2 pathway for all cold-induced genes analyzed as shown in Table 6. Collectively, these results indicate circadian clock genes are notably affected by cold shock.

A group of circadian clock genes, and their associated miRNAs were upregulated upon cold shock (Tables 7 and 8). Similar upregulation of circadian clock genes by cold stress have also been observed in previous studies [15, 38]. It suggests a possible involvement of circadian associated regulation during cold acclimation and cold shock. However, direct evidence for a role of circadian clock genes in cold responses is still lacking. Circadian rhythm controls the daily rhythm for an organism in behavior, physiology and biochemistry [40]. The molecular basis of this oscillation consists of interaction between positive and negative feedback loops. At the molecular level, the circadian rhythm is governed by a set of transcription activators, CLOCK and NPAS2/BMAL1, which induce expression of repressors, PER1-3/CRY1-2, to form an interacting feedback loop [49]. BHLHE41 functions to inhibit CLOCK:BMAL1 transactivation of the clock gene *Per1* to resembles the negative feedback loop of PER/CRY complex [41]. In addition to light, circadian rhythm could also be entrained by temperature and feeding behavior [50]. Zebrafish core clock genes, including *cry2a*, *cry3* and *per4*, can be entrained by lower temperature but not *per2* [51]. This implies the elevation of *per2* in our study may play a novel biological function in the cold stress response. Here, we successfully demonstrate that overexpression of core clock gene *per2* can increase cold tolerance in zebrafish larvae upon cold shock, but not *bmal*. Previous studies showed that knockout *per2* impairs gluconeogenesis [52] but *bmal* null mice increased gluconeogenesis [53], suggesting two proteins may play opposite role in glucose metabolism. The *per2* gene may have a glucocorticoid response element that responds to glucocorticoid hormones to increase blood glucose [54]. The elevation in plasma glucose is enhanced by the glucocorticoid/cortisol-mediated gluconeogenesis, which is a common stress response in aquatic creatures in response to cold [55]. We observed the basal glucose level remains high in transgenic zebrafish larvae overexpressing *per2*, which suggests that the elevation of *per2* expression level may counter cold stress by maintaining a higher gluconeogenesis. Per2 is also known to control lipid metabolism by direct regulation of PPARγ [56]. Whether Per2 also exerts its cold-defending function via enhanced lipid metabolism still awaits further investigation. Furthermore*, bhlhe41*-overexpressing fish also showed better cold tolerance in locomotor activity and retain higher glucose level. It will be interesting to study whether the inhibition of CLOCK/BMAL complex would enhance cold tolerance.

miRNAs play a key role in cellular responses to the changing environment. Upon stress, cells also tend to restore or reprogram their transcriptomes to counter the changes modulated by miRNAs [27]. It implies a plausible role of miRNAs in mediating cold responses. However, Yang et al. previously reported that cold acclimation trigger changes in some miRNA profiles in adult zebrafish brains and those miRNAs have poor associations to the affected genes. They concluded that microRNAs play a minor role in mediating transcriptome plasticity during cold stress [30]. It appears to be contradictory to our finding that the affected miRNAs have predicted target genes involved in cold-inducible biological processes, including melanogenesis, GnRH signaling pathway and circadian rhythm.

To clarify the interaction between *per2* and associated *dre-mir-29b*, we examined their dynamic expression during cold shock and found good correlations for *dre-mir-29b* to serve as an inhibitor of *per2* expression. The dynamic pattern between miRNAs and target genes nicely matches the “pulse model” as described by Leung et al. [27]. As miRNAs accumulate over time, miRNAs exert their suppressive effect on the mRNA targets, causing a decrease in mRNA target expression. The poor correlation between cold-affected miRNAs and mRNAs observed previously [30] might be due to the time of sample collection or the use of different tissues (brain tissue vs. whole larvae used here). The duration of stress also determines if cells can re-establish homeostasis or initiate the mechanism to acclimate to the new environment [57]. The discrepancy between two studies may also be resulted from different machineries used in two different stress levels (cold acclimation v.s. cold shock).

## Conclusions

Here, we demonstrate for the first time that miRNAs can be fine turners for regulating genomic plasticity against cold shock. We further showed that the fine tuning of core clock gene *per2* via its associated miRNA, *dre-mir-29b*, can enhance the cold tolerance of zebrafish larvae. The circadian clock genes may thus serve as targets for designing novel strategies to face cold challenges in aquaculture.

## Additional files

Additional file 1: Lists of primers. (XLSX 10 kb)
Additional file 2: Reads length, chromosome distribution and BCA analysis of smRNA-seq. (DOCX 2284 kb)
Additional file 3: Heat maps of cold-related miRNAs and genes. (XLSX 105 kb)
Additional file 4: Lists of cold-related miRNAs and target genes. (XLSX 530 kb)
Additional file 5: GO term analysis of miRNA corresponding target genes. (XLSX 135 kb)
Additional file 6: List of cold-related genes and GO term analysis. (XLSX 30 kb)
Additional file 7: Expression profiles of miRNAs and their target mRNAs. (XLSX 104 kb)
Additional file 8: KEGG pathway analysis of dre-mir-29b and genes involved in the regulation of RNA metabolic process. (XLSX 17 kb)

## Abbreviations

3′ UTR: 3′ untranslated region
*arntl1a*: Aryl hydrocarbon receptor nuclear translocator-like 1a
*bhlhe41*: Basic helix-loop-helix family, member e41
*cirbp*: Cold inducible RNA binding protein
DAVID: The Database for Annotation, Visualization and Integrated Discovery
Dpf: Days post fertilization
Dre: *Danio rerio*
F: Forward
GnRH: Gonadotropin releasing hormone
GO: Gene ontology
H2A: Histone 2A
hpf: h post fertilization
hpi: h post induction
KEGG: Kyoto Encyclopedia of Genes and Genomes
miRNAs: MicroRNAs
MO: Morpholino oligonucleotides
NOD: Oligomerization domain
Nts: Nucleotides
Per2: Period 2
qPCR: Quantitative polymerase chain reaction
R: Reverse
RIG-I: Retinoic acid-inducible gene 1
RPKM: Reads per kilo base per million
UTR: Untranslated region
