# Supplementary material for: MicroRNAs regulate gene plasticity during cold shock in zebrafish larvae
Source: BMC Genomics. 2016 Nov 15;17:922. doi: 10.1186/s12864-016-3239-4 (PMC5111229; doi:10.1186/s12864-016-3239-4)
Supplement: Additional file 2: — Reads length, chromosome distribution and BCA analysis of smRNA-seq. (DOCX 2284 kb) [file 12864_2016_3239_MOESM2_ESM.docx]

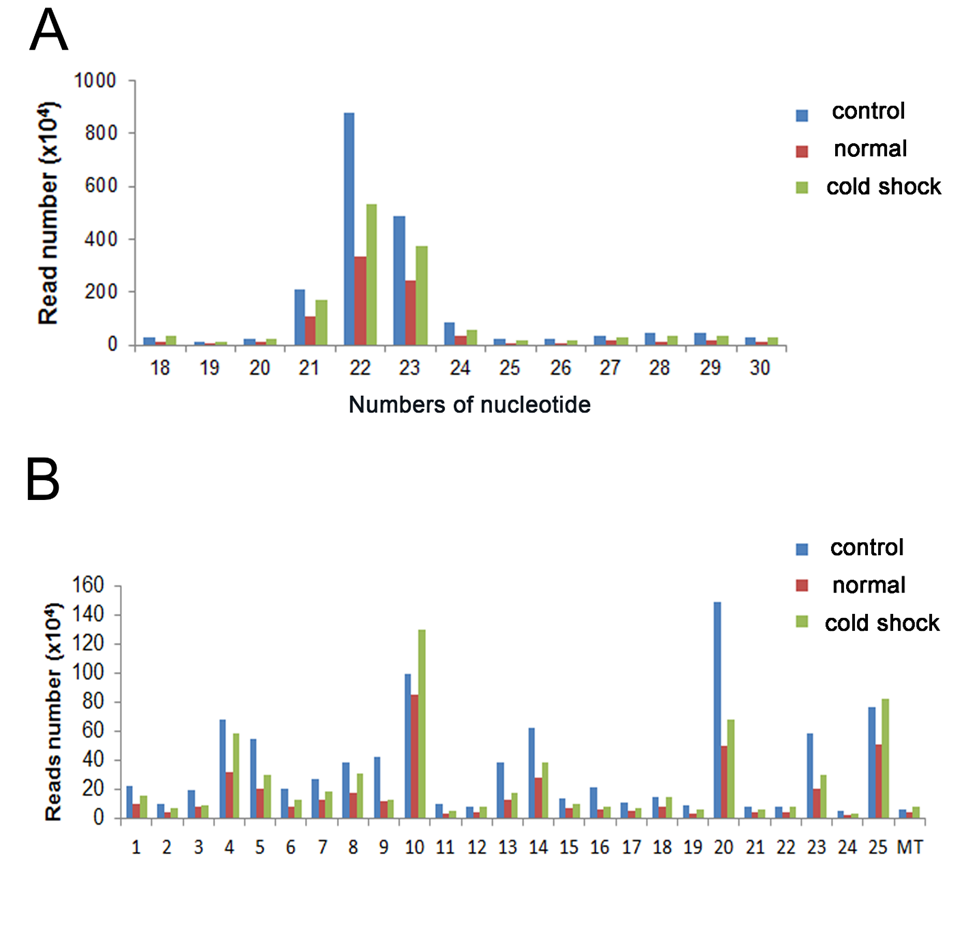


Additional file 1: Figure S1: Length (A) and chromosome (B) distribution of small RNA reads. RNA reads from the control and treatments are in different colors as designated. Number on X axis indicate the number of chromosome. MT: mitochondria


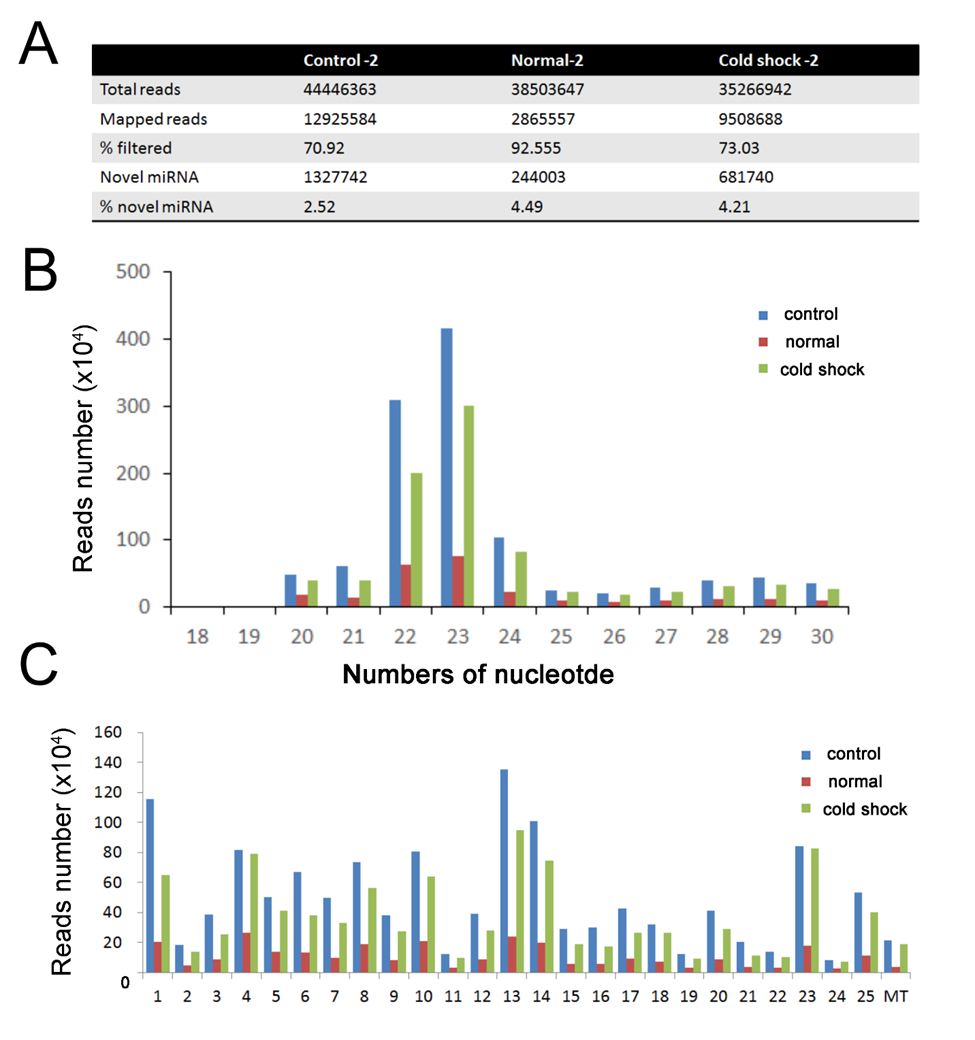


Additional file 1: Figure S2: Quality profile of smRNA-seq data (A)Total reads, mapped reads and novel miRNA reads of each sample are shown. Length (B) and chromosome (C) distribution of small RNA reads. RNA reads from the control and treatments are in different colors as designated. Number on X axis indicate the number of chromosome. MT: mitochondria


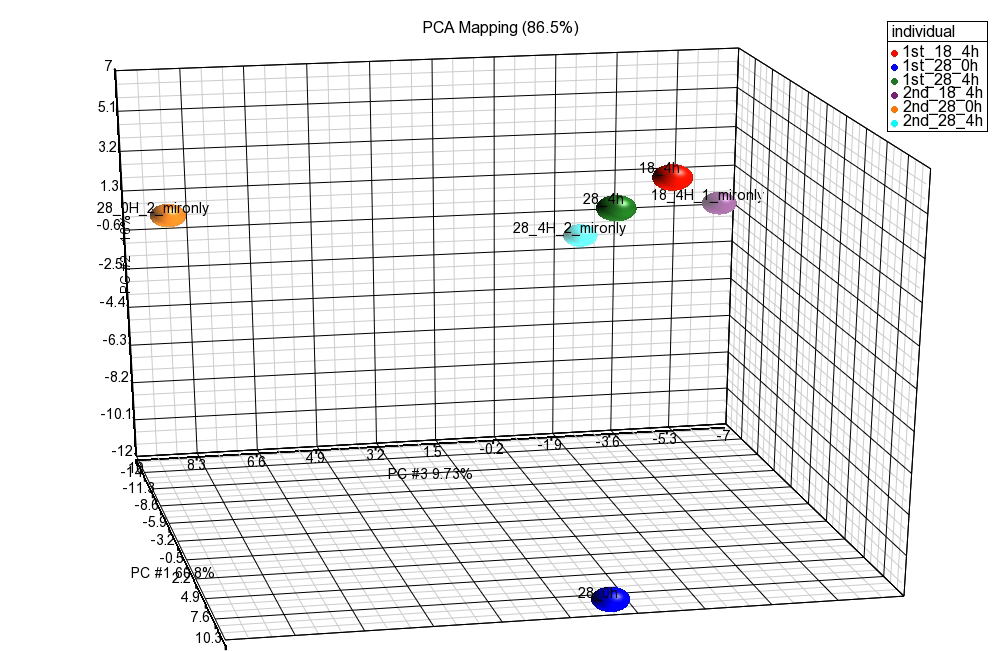


Figure S3 : Principle component analysis (PCA) of miRNA profiles. Duplicate samples from three groups were subjected to PCA using Partek. The x-, y- and z-axes represent PC1, PC2 and PC3, respectively. The colors points indicate temperature treatment and time of exposure of different batches. The sample names were displayed within the figure.
